# Prognostic Value of Electrical Impedance Spectroscopy (EIS) When Used as an Adjunct to Colposcopy – A Longitudinal Study

**DOI:** 10.2478/joeb-2020-0012

**Published:** 2020-11-06

**Authors:** B. H. Brown, P.E. Highfield, J. A. Tidy

**Affiliations:** 1 The University of Sheffield, Sheffield, UK; 2 Technical consultant, Manchester, UK.; 3 Department of Gynaecological Oncology, Sheffield Teaching Hospitals, NHS Foundation Trust, Sheffield, UK

**Keywords:** Bioimpedance, cervical, cancer, impedance, spectroscopy, prognosis, colposcopy

## Abstract

**Objective:**

Colposcopy can be used with Electrical Impedance Spectroscopy (EIS) as an adjunct, to assess the presence of High Grade Cervical Intra-epithelial Neoplasia (CIN2+). This analysis of longitudinal data has used the results from women with a negative colposcopy, in order to see if the initial (index) EIS results were able to predict the women who subsequently developed CIN2+. A further objective was to investigate what tissue structural changes might be reflected in the electrical impedance spectra.

**Methods:**

847 patients were referred with low grade cytologly. EIS measurements were made around the transformation zone of the cervix during colposcopy. Every EIS spectrum was matched to a template representing CIN2+ and the result was positive if the match exceeded a probability index threshold. The colposcopic impression was also recorded. All the women who developed biopsy proven CIN2+ within three years of the index colposcopy were identified.

**Results:**

The median follow-up was 30.5 months. Where both CI and EIS were initially positive, there was an increased prevalence (8.13%) of CIN2+ developing as opposed to 3.45% in the remaining patients (p=0.0159). In addition, if three or more EIS spectra were positive there was a higher prevalence (9.62% as opposed to 3.56% p=0.0132) of CIN2+ at three years. The index spectra recorded from the women who developed CIN2+ showed EIS changes consistent with increases in the extracellular volume and in cell size inhomogeneity.

**Conclusion:**

EIS does offer prognostic information on the risk of CIN2+ developing over the three-year period following the EIS measurements. The changes in EIS spectra are consistent with an increase in cell size diversity as pre-malignancy develops. These changes may be a consequence of increased genetic diversity as neoplasia develops.

## Introduction

Colposcopy is widely used as part of screening programmes to detect cervical cancer and pre-cancer. The main objective of colposcopy is usually the detection of high-grade disease, defined as cervical intra-epithelial-neoplasia grade 2+ (CIN2+). There are a wide range of published diagnostic accuracy figures for clinical colposcopy. For example, Mitchell [1] in 1998 reported from 8 studies, as part of a meta-analysis, a weighted mean sensitivity of 85% and specificity 69%. Underwood [2] in 2012 reported on 25 studies and give pooled values of 91.3% for sensitivity and 24.6% for specificity. Both Mitchell and Underwood reported a very wide variation in diagnostic accuracy figures. Brown and Tidy [3] in 2019 reviewed 18 publications that included sufficient raw data to enable comparative diagnostic accuracy figures to be calculated. They reported a weighted mean value of 75.1% for the sensitivity of colposcopy. These figures for sensitivity suggest that there are a significant number of false negative results from colposcopy. The recent changes in many organised cervical screening programmes, such as the assessment of primary human papillomavirus (HPV), has increased the number of women referred to colposcopy at very low risk of CIN2+. As a result, the poor performance of colposcopy is often a concern to colposcopists when discharging women from clinic after a negative colposcopy. The use of adjuncts in colposcopy, such as ZedScan^TM^ has been shown to increase the detection of CIN2+ [4]. However, long term outcome data from women who have been discharged to community-based screening is lacking.

This current investigation reports the results of a longitudinal service evaluation of patients who were negative at the initial (index) colposcopy but who, in some cases, were found to develop CIN2+ over the subsequent three years. The investigation also looked for any association between the index Electrical Impedance Spectroscopy (EIS) measurements and the subsequent development of disease.

## Methods

Women who underwent an EIS examination using ZedScan^TM^, and had a negative colposcopic examination, were extracted from an on-going service evaluation of women referred for colposcopy during the period of December 2013 to August 2018. Women with a high-grade cytology referral were excluded. In addition, women referred following a previous colposcopy and women with clinical symptoms were excluded. Colposcopies were performed at the Jessop Wing Colposcopy Unit, Sheffield, UK. The cervical screening programme in Sheffield was one of six laboratories contributing to the NHS cervical screening evaluation of primary HPV screening (hrHPV) [5]. In every case the results of the index colposcopy examination were considered negative for the presence of high grade cervical intra-epithelial neoplasia (CIN2+), if the colposcopic impression (CI) was less than CIN2+ and if either, no biopsy was taken or, any biopsy taken was reported as CIN1 or less. Women with a CI of CIN2+ underwent biopsy. All the women were the subjects of a follow-up evaluation from the time of the index colposcopy. Women who developed biopsy proven CIN2+ within the three-year period were identified from the cytology, colposcopy and histopathology hospital databases.

The outcomes measured included the subsequent prevalence of CIN2+ and, to allow for differences in the duration of follow-up between patients, the annual risk of developing CIN2+, expressed as a percentage risk. By using the actual time from the index colposcopy to the detection of disease in each woman this takes into account the fact that these times are not randomly or uniformly scattered within the three years.

This was a service evaluation carried out in the Jessop Wing Colposcopy clinic and so no ethical approval was required [6]. All patient data was anonymised.

**Figure 1:**
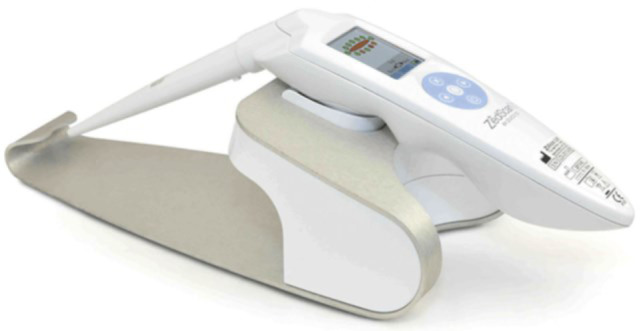
The ZedScan^TM^ handset, used to make the EIS measurements. The handset is shown placed on the base. The tapered disposable is shown on the left and terminates in the four AgCl electrodes that are placed on the tissue. The screen is used to guide the clinician to take up to 12 measurements around the transformation zone of the cervix.

The index colposcopy examination included the measurement of EIS as an adjunct to the examination. The outcome included both the Colposcopic Impression (CI) and the result of the EIS measurements. Since February 2013 the colposcopy unit has used EIS, as an adjunct to colposcopy to help increase detection of CIN2+^4^. EIS spectral measurements were made, using ZedScan^TM^, from a maximum of 12 points (minimum number was 9) around the transformation zone between the squamous and columnar cervical epithelia. Each measurement was of the complex transfer impedance between the pairs of current drive and receive electrodes that were on the tip of a hand-held probe, that was placed in contact with the tissue (see Fig 1). Only the real part of the complex impedances has been used in this analysis. The impedances were measured at 14 frequencies, logarithmically spaced between 76 Hz and 625 kHz. The disposable tip of ZedScan^TM^ includes four AgCl electrodes of diameter 0.6 mm, with centres spaced around a circle of 2 mm diameter. This spacing of electrodes was chosen in order to be sensitive to tissue changes in cervical epithelium to a depth of about 400 μm. Several quality control checks are made on the measured impedance spectra before data is stored.

When used as an adjunct to colposcopy, ZedScan^TM^ determines an index of probability that a measured spectrum corresponds to the presence of CIN2+. This index value is determined by calculating the match to a stored template that was determined from 3-D finite element, cellular-level, modelling of the structure of high grade CIN epithelium. This structure was determined by measurements made from histopathology images of diseased tissue [7,8]. The calculated index of probability is used to guide the clinician in taking a diagnostic biopsy. In the current study an index threshold of 0.87, derived from previous studies for low grade referrals, was used for all the patients where the CI was negative. A lower threshold was applied where the CI was positive [4]. In every case the number of measured spectra that were above these thresholds was recorded, as occurring at either one, two or greater than two points. The outcome of the index EIS examination was recorded as positive if at least one of the twelve measured spectra was above the threshold. All the results were placed in one of four groups defined by both CI and EIS being negative, CI being negative but EIS positive, both CI and EIS being positive or CI being positive but EIS negative.

The measured spectra were also placed into two groups according to whether or not CIN2+ developed over the three-year follow-up period. The EIS spectra were then inspected to determine any significant differences between the two groups. Comparisons between the group means were made using an “N-1” Chi-squared test, as recommended by Campbell [9] and Richardson [10]. The Yates correction was used to allow for the small numbers in the groups. A Mann-Whitney non-parametric U test was also used to compare the groups.

EIS spectra always fall with increasing frequency. The reason for this fall in impedance is that tissue consists of components with both electrically resistive and capacitive properties, determined by the presence of ions, some of which are bound in cell membranes and others free to move. If a potential is applied to tissue the capacitive components will store charge. When the potential is removed, the capacitors will discharge through the resistive components. Debye [11] showed that if this discharge is exponential then equations can be derived that show how impedance will fall with increasing frequency. If the Debye calculated spectra are compared with measurements from tissue it is found that the agreement between the theoretical and measured values is relatively poor. Cole and Cole [12] proposed a modification to the Debye equations that included a term Alpha. Values for Alpha can be used to give a good fit to measurements made on many tissues. The Cole equation can be written in terms of the resistive component of impedance (*Z*) as follows:

$$Z=R_{\infty }+\frac{\left(R_{0}-R_{\infty }\right)}{(1+{\left(jF/F_{c}\right)}^{\left(1-\alpha \right)}}$$

In this equation *R*_0_ and *R*_∞_ are the resistances at zero and infinite frequency, respectively. *F* is the frequency of measurement. *F*_c_ is often referred to as the characteristic frequency. If a tissue, such as blood, is composed of a suspension of cells all of very similar size, then *F*_c_ increases as cell sizes decrease. It can also be shown that if the cell sizes are not homogeneous, but have a distribution of sizes, then *α* will increase. A population of cells is often referred to as a dispersion.

A commonly used interpretation of the four Cole equation parameters for tissue is that the inverse of extracellular volume determines *R*_0_, the inverse of the total volume determines *R_∞_*, cell sizes determine *F*_c_, which is the centre of the dispersion, and *α* is determined by the inhomogeneity of the cells within the dispersion. The Cole equation is an empirical equation that does not offer any quantitative figures that can be directly associated with tissue properties. The conductivity of the intracellular and extracellular spaces will also affect both *R_0_* and *R_∞_*. See Grimnes and Martinsen for a discussion of the use of the Cole equation [13].

**Figure 2:**
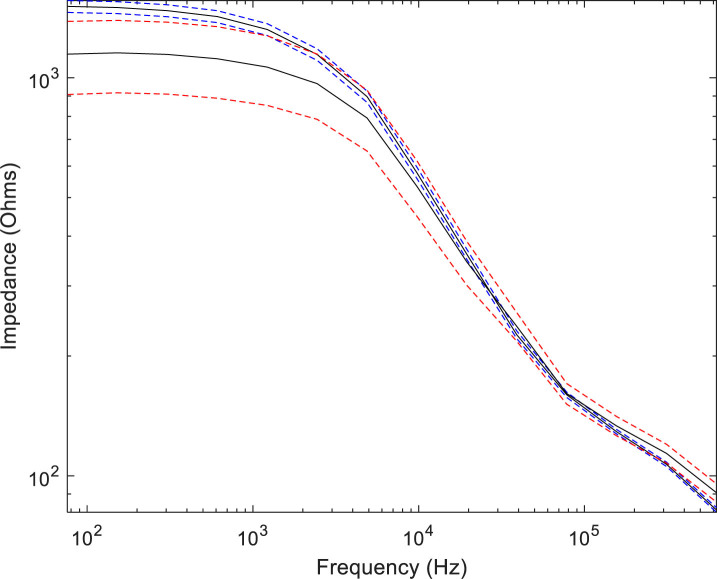
This shows the median of all the 31 spectra where CIN2+ occurred in black with the 95% confidence levels shown in red. The median of all the 536 spectra where CIN2+ did not occur is also shown in black, but with the 95% confidence levels shown in blue. Log scales are used on both axes.

In order to look for any changes in *F*_c_ or *α,* ideally the Cole equation should be fitted to every measured spectrum. Unfortunately, this was not possible because the individual spectra contained some noise and did not all show a clear dispersion, such as those shown in the mean spectra of Fig 2. Two alternative approaches were used to look for changes in *F*_c_ and *α*. In the first, the slope of the spectrum between frequencies 1.22 and 2.44 kHz was determined, as a proxy for *α*. In the second, the Cole equation was fitted to the overall mean spectra for the two groups of women who either did or did not develop CIN2+.

### Ethical approval

The research related to human use has been complied with all relevant national regulations, institutional policies and in accordance with the tenets of the Helsinki Declaration, and has been approved by the authors’ institutional review board or equivalent committee.

## Results

847 women who met the evaluation criteria were identified. Of these, 579 women were referred with low grade abnormalities (Borderline nuclear change in squamous cells (ASCUS) or low grade dyskaryosis (LSIL)) and 268 women with persistent hrHPVpositive / cytology negative results.

The median time of follow-up for all the patients, to the point where CIN2+ was identified, was 30.5 months. The patients were grouped as shown in Table 1. The median was 35 months for Group 1, 34 months for Group 2 and 19 months for Group 3. The prevalence of CIN2+ at 3 years for the evaluated population was 4.13% and the risk of developing CIN2+ at 3 years was 1.69% per annum (pa) over the follow-up period. The prevalence of CIN2+ and the risk of developing CIN2+ varied according to the outcome of the index colposcopic and ZedScan examinations (Table 1).

**Table 1:** This shows how the 847 women were grouped for analysis. The prevalence of CIN2+, and the annual risk of disease, is also given for each group. The annual risk takes into account the fact that the first three groups of women did not have the same median period from the time of the index colposcopy to the time when disease was detected.

	**Outcome of colposcopy (CI) and EIS**	**Prevalence of CIN2+ within 3 years**	**Percentage risk of developing CIN2+ per annum, over the follow-up period for each woman**
**Group 1** **396 women**	Both CI and EIS negative **(Double negative)**	CIN2+ arose in 13 (3.28%)	1.20%
**Group 2** **318 women**	CI negative but EIS positive	CIN2+ arose in 12 (3.77%)	1.64%
**Group 3** **123 women**	Both CI and EIS positive **(Double positive)**	CIN2+ arose in 10 (8.13%)	4.57%
**Group 4** **10 women**	CI positive but EIS negative	No cases of CIN2+	N/A

The only one of the four groups that is significantly different from the other groups, in terms of the prevalence of CIN2+ arising in the three years following the index colposcopy, is group 3. Group 3 is associated with an increased prevalence (8.13%) of CIN2+ over the three years following the index colposcopy when compared with the other three groups combined (3.45%, p=0.0159). Group 3 is also associated with an increased prevalence when compared with groups 1 and 4 combined, where the EIS outcome was negative (3.2%, p=0.019).

The EIS spectra were also inspected to see if the number of EIS positive spectra recorded in each woman was correlated with the prevalence of CIN2+. These figures were determined only for women where EIS was positive. The total number of women in this group is 441. The incidence of disease in these 441 women was broken down on the basis of the number of positive EIS spectra (Table 2).

**Table 2:** The breakdown of the prevalence of disease in women where the index CI was negative and at least one EIS point was above the higher probability index threshold of 0.87.

**Number of abnormal EIS points**	**Number of women**	**Cases of CIN2+ within three years**	**Prevalence of disease**
**One**	226	7	3.1%
**Two**	111	5	4.50%
**Three or more**	104	10	9.62%
**One or two**	337	12	3.56%

The group of 104 women where three or more spectra were above the probability index threshold, was significantly higher than in the remaining women (p= 0.0132).

In order to compare the spectral shapes in women who either did or did not develop CIN2+ the overall median spectra for 31 women who did develop CIN2+ and 536 women who did not develop CIN2+ over the three-year follow-up were determined. See Fig 2.

**Figure 3:**
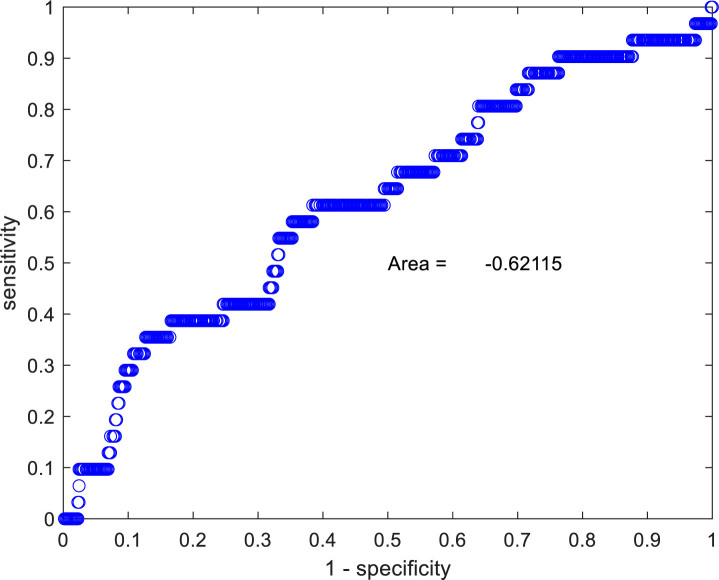
An ROC curve comparing the EIS impedance of the two groups, at a frequency of 152 Hz, who either did or did not develop CIN2+. The AUC is 0.621, p=0.0088 when using a Mann-Whitney U test.

The difference between the mean spectra is most marked at the lowest and highest frequencies. Application of an EIS cut-off of <800 Ohms at a frequency of 152 Hz identifies 16.6% (89/536) of the women in whom CIN2+ did not arise and 38.7% (12/31) in those where CIN2+ did arise. These figures correspond to a sensitivity of 38.7% and specificity of 83.4%. The results for the two groups at 152 Hz are statistically significantly different (p=0.0018). An ROC curve using the measured impedance at 152Hz is shown in Fig 3. The area under the curve (AUC) is 0.621. A Mann-Whitney non-parametric U two-tailed test gives p=0.0088 when comparing the AUC of the two patient groups.

The differences between the two groups that did, or did not, develop CIN2+, at the lower EIS frequencies may be explained by a larger extracellular volume in the women who developed CIN2+. The changes at the highest frequencies may be the result of intracellular changes.

The slope of the EIS spectra between frequencies 1.22 and 2.44 kHz was used as a proxy for *α*. An ROC curve used to compare the groups who either did or did not develop CIN2+ gave an area-under-the-curve (AUC) of 0.596. This shows a weaker relation between the measured slope and the development of CIN2+. Selection of a cut-off of −0.080 to the data gives a sensitivity of 45.2% (95% CI 27.8–63.7) and specificity 70.1% (95% CI 66.0–74.0).

The Cole equation parameters for the two mean spectra shown in Fig 2 are given in Table 3. The Cole equation was fitted to the spectra using least-square minimisation.

**Table 3:** The Cole equation parameters of the curves shown in Fig 1.

Women who did not develop CIN2+	R_o_ = 1485	*R*_∞_ = 80	F_c_ = 4500	*α* = 0.18
Women who did develop CIN2+	R_o_ = 1220	*R*_∞_ = 90	F_c_ = 5100	*α* = 0.24

It is not possible to put error figures to this data but it does appear that both *F*c and *α* are increased in the women who develop CIN2+.

**Figure 4:**
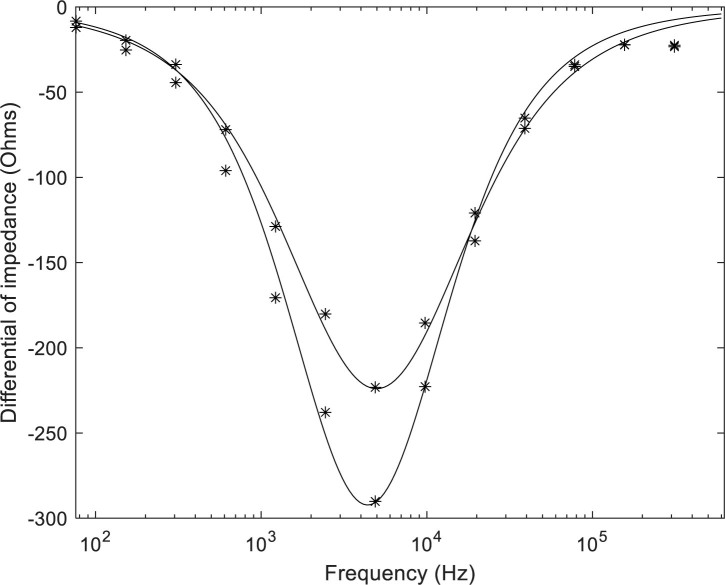
Comparison of the frequency differential of the impedance spectra for the CIN2+ women (upper curve) and the <CIN2+ women (lower curve). The curves are from the fitted Cole equation. The parameters of the equation are given in Table 3.

Fig 4 shows the frequency differential of the two mean spectra shown in Fig 2. The points show the measured differentials, and the solid lines show the fitted Cole equations. It can be seen that the Cole equations fit the measured data quite well. It also shows the difference in the spectral shapes between the groups who did and did not develop CIN2+. The spectrum from the group who developed CIN2+ shows a smaller peak gradient and also a wider dispersion. This is consistent with an increase in *α*.

## Discussion

The main objective of this study was to use colposcopy and the associated EIS data collected over more than five years to see if the initial (index) results were able to identify any increased risk of CIN2+ developing over a three-year follow-up. All the women had a negative outcome following the index colposcopy. The results show that women where both the colposcopic impression (CI) and the EIS analysis were positive, even though no CIN2+ was identified, had an increased prevalence of CIN2+ over the subsequent three years. The prevalence was 8.13% as opposed to 3.45% in the remaining women. The risk of CIN2+ developing in the whole group was 4.13%. This figure is consistent with that reported by Kelly *et al.* [14], who reported on a follow-up of 965 women, where it was found that 4.4% developed CIN2+ over a median time of follow-up of 27 months. Cruickshank *et al.* [15], in a study of 884 women, recorded that 4.86% developed CIN2+ over a mean length of follow-up of 31 months. They also showed that 8.58% of the women who were hrHPV positive, this group being similar to our group, developed CIN2+ with a risk of 3.3% pa. We have demonstrated in this study that, where colposcopy and EIS results were negative, both the prevalence and risk of developing CIN2+ are further reduced (3.28% and 1.20% pa).

EIS, when used as an adjunct to colposcopy, identifies points on the cervix where the EIS spectrum is consistent with the presence of CIN2+. This information is used to guide the clinician as to where best to take a biopsy. In this study we compared the incidence of disease in women where three or more points were identified as opposed to fewer such points. It was found that the women where three or more points were identified had a higher incidence of CIN2+ (9.62%) over the subsequent three years. This result is consistent with EIS identifying an increased spatial spread of pre-malignant changes in these women.

The EIS data was also analysed in isolation to see if this was able to identify the increased risk of CIN2+ developing. It was found that the group of women who developed CIN2+ had a significantly lower impedance at the lower EIS frequencies (p=0.0088). There also appeared to be changes in the shape of the EIS spectra in the EIS frequencies around 1–10 kHz. The characteristic frequency and width of the dispersion appeared to be changed. These changes may be interpreted as arising from increases in both extracellular volume and tissue inhomogeneity in the women who developed CIN2+.

A weakness of this study is that, whilst the data is taken from a relatively large group of women, the numbers who developed CIN2+ are quite small. The results do reach statistical significance but should none-the-less be looked at critically in future research.

It is interesting to speculate as to the reasons why the changes we observed in the EIS spectra were associated with an increased risk of CIN2+ in some women. Can a prognostic value, based upon EIS, be explained in terms of an increased diversity of cellular structures? Increased diversity of cell sizes, and shapes should increase the dispersion in an EIS spectrum and will also change the architecture of the extracellular space. A homogeneous cell population can be closely packed with a relatively low extracellular volume. However, if cells are less homogeneous in size then they cannot be closely packed and extracellular volume will be larger. Possibly the genetic diversity of pre-cancer cells is giving rise to EIS changes, and the presence of these changes over several sites around the transformation zone, is evidence of this increased diversity? These changes would not be apparent when screening cytology or histopathological examination of biopsied epithelia are being assessed.

Wilson [16] pointed out a number of publications reporting the genetic diversity associated with the progression of bladder cancer [17] and Barrett's oesophagus [18]. Maley *et al.* [19] reviews the evolutionary features of neoplasms. Multiple biopsy samples from neoplasms in one individual show a very wide genetic diversity. It is possible that gene expression then leads to an increasing inhomogeneity of cell structures as cancers evolve.

The main conclusion of this study is that EIS does appear to contain prognostic information on evolving cervical neoplasia. This should be useful in patient management following a negative colposcopy. This management would be different for the patient groups shown in Table 1. If both colposcopy and EIS are negative, then these women are at a very low risk of developing CIN2+ at three years. However, if EIS changes are present then these can be used to assess any increased future risk. A further significant conclusion is that the changes in EIS spectra in women who develop high grade CIN are consistent with an increase in the diversity of cell sizes and extracellular volume in the epithelium. This may be a consequence of increasing cellular diversity as cancer evolves.
